# Case report of a 28-year-old man with aortic dissection and pulmonary shadow due to granulomatosis with polyangiitis

**DOI:** 10.1186/s12890-019-0884-9

**Published:** 2019-07-08

**Authors:** Lei Pan, Jun-Hong Yan, Fu-Quan Gao, Hong Li, Sha-Sha Han, Guo-Hong Cao, Chang-Jun Lv, Xiao-Zhi Wang

**Affiliations:** 1 Department of Respiratory and Critical Care Medicine, Binzhou Medical University Hospital, 661 Yellow River Road, Binzhou, 256603 China; 2 Department of Ultrasonography, Binzhou Medical University Hospital, Binzhou, 256603 China; 3 Department of Pathology, Binzhou Medical University Hospital, Binzhou, 256603 China

**Keywords:** Granulomatosis with polyangiitis, Vasculitis, Aortitis, Aortic dissection

## Abstract

**Background:**

Granulomatosis with polyangiitis (GPA) is characterised by the main violation of the upper and lower respiratory tract and kidney. GPA is considered a systemic vasculitis of medium-sized and small blood vessels where aortic involvement is extremely rare.

**Case presentation:**

A 28-year-old male was admitted to the hospital due to 4 h of chest pain. Computed tomography scan of the aorta showed a thickened aortic wall, pulmonary lesions, bilateral pleural effusion and pericardial effusion. The aortic dissection should be considered. An emergency operation was performed on the patient. Surgical biopsies obtained from the aortic wall showed destructive changes, visible necrosis, granulation tissue hyperplasia and a large number of acute and chronic inflammatory cells. Nearly a year later, the patient was re-examined for significant pulmonary lesions. His laboratory studies were significantly positive for anti-neutrophilic antibody directed against proteinase 3. Finally, the diagnosis of GPA was obviously established.

**Conclusions:**

Although GPA rarely involves the aorta, we did not ignore the fact that GPA may involve large blood vessels. In addition, GPA should be included in the systemic vasculitis that can give rise to aortitis and even aortic dissection.

**Electronic supplementary material:**

The online version of this article (10.1186/s12890-019-0884-9) contains supplementary material, which is available to authorized users.

## Background

Granulomatosis with polyangiitis (GPA), formerly known as Wegener’s granulomatosis, is characterised by the main violation of the upper and lower respiratory tract and kidney [1] .In fact, the disease was first reported by Peter McBride in 1897 [2] and was completely described by Wegener in 1936 [3]. GPA is one of the anti-neutrophil cytoplasm antibody (ANCA)-associated systemic vasculitis of medium-sized and small blood vessels [4–6]. The incidence rate of GPA is 10–20 cases per million per year [7]. GPA can occur at any age but most often between the ages of 40 and 65.Aside from the lungs and kidneys, GPA can also affect the joints, eyes, ears, skin and heart. Pericarditis and coronary vasculitis are the most frequent findings of cardiac involvement in GPA [8]. Although GPA can accumulate large blood vessels, it is very rare in clinical practice. Aortic involvement, such as aortic dissection, is an even more rare presentation in GPA. In our case report, we describe aortic dissection of the ascending aorta and aortic arch as the first manifestation of GPA in a 28-year-old male patient.

## Case presentation

**Fig. 1:**
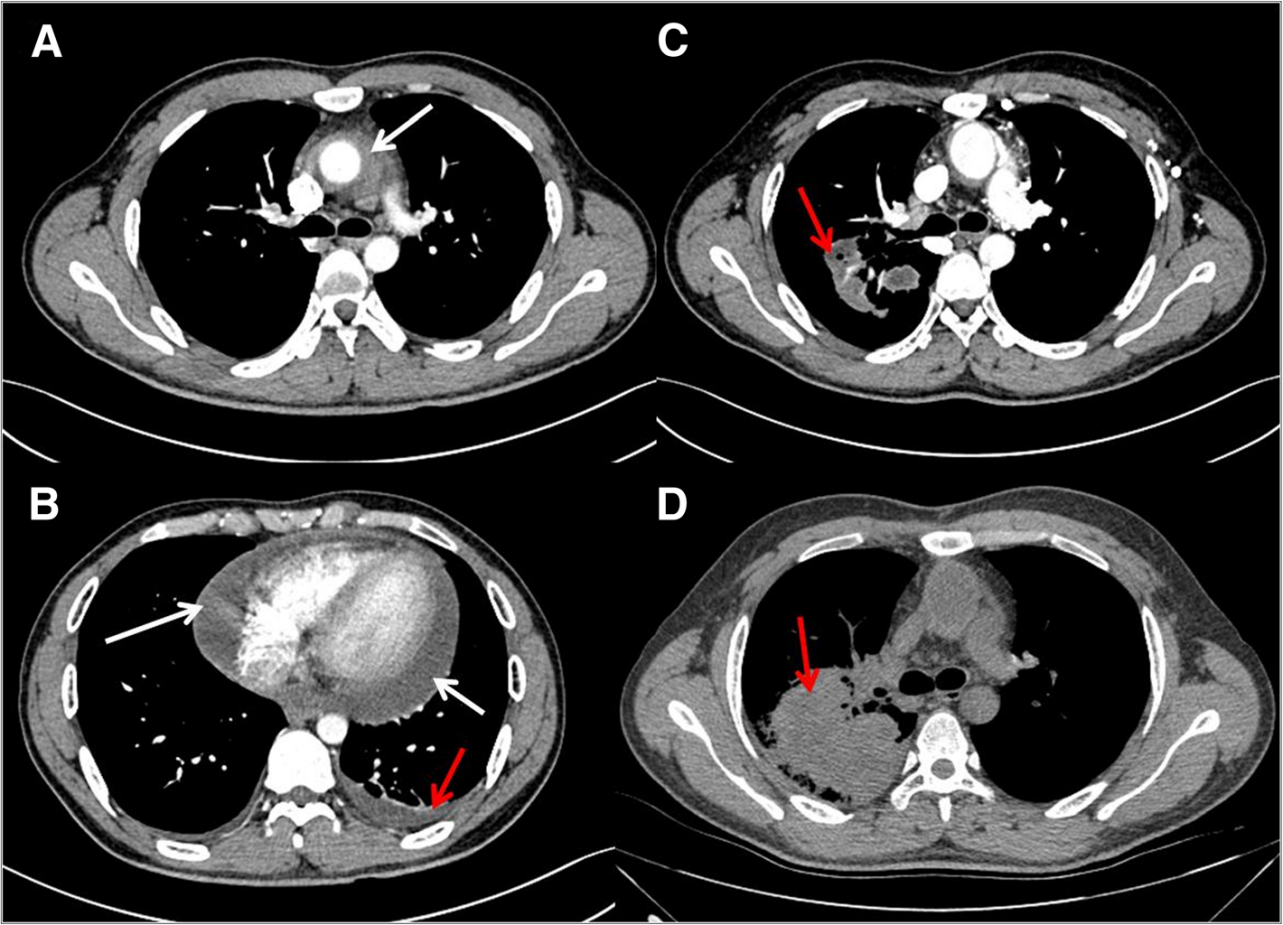
Computed tomography scan of the aorta shows a thickened aortic wall, pulmonary lesions, bilateral pleural effusion, and pericardial effusion: **a** Ascending aortic dissection and thickened aortic wall (white arrow); **b** Bilateral pleural effusion (red arrow) and pericardial effusion (white arrow); **c** pulmonary lesions (red arrow); **d** pulmonary lesions (red arrow)

**Fig. 2:**
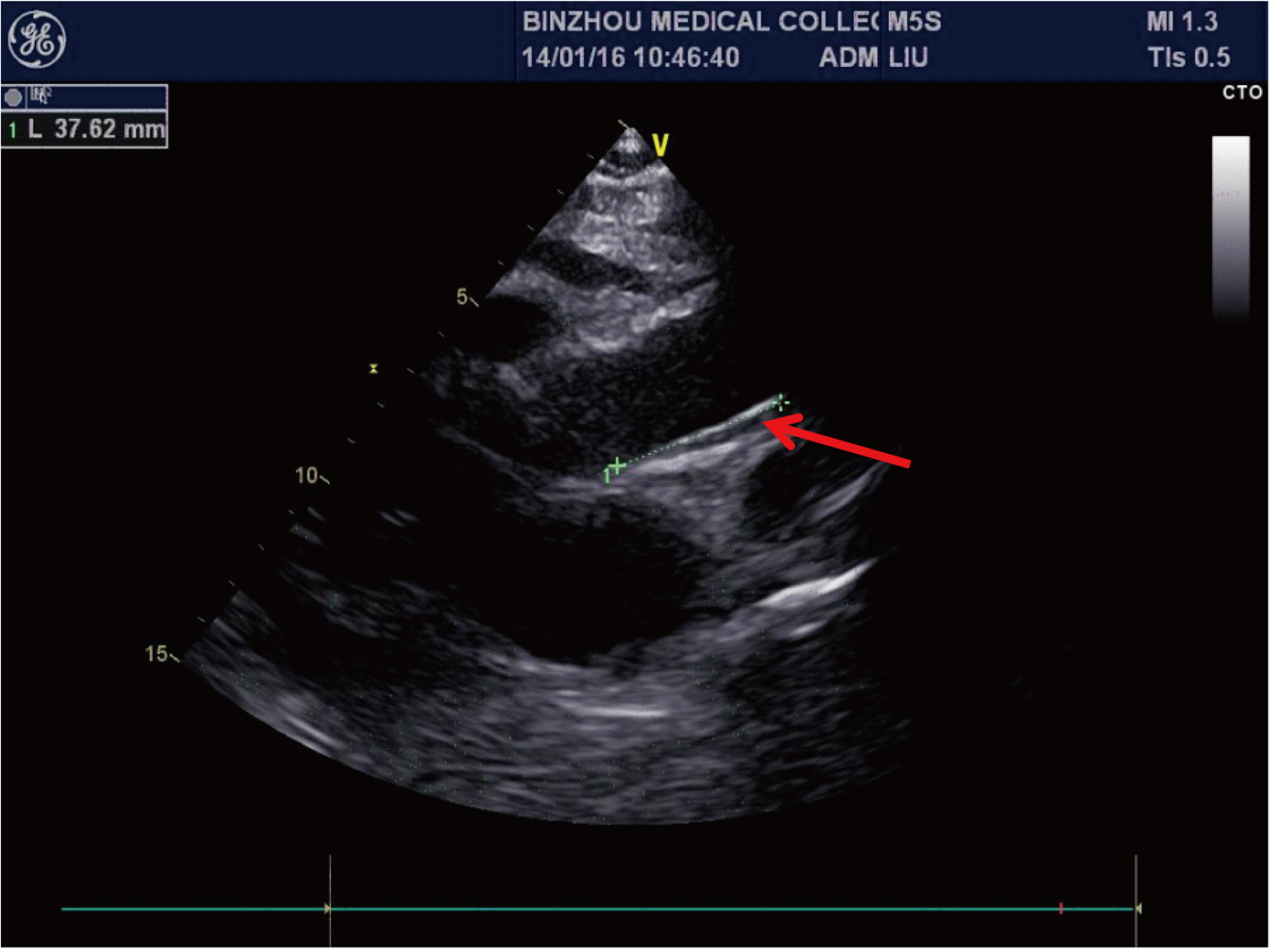
Cardiac colour ultrasound suggesting aortic hematoma or dissection (red arrow)

**Fig. 3:**
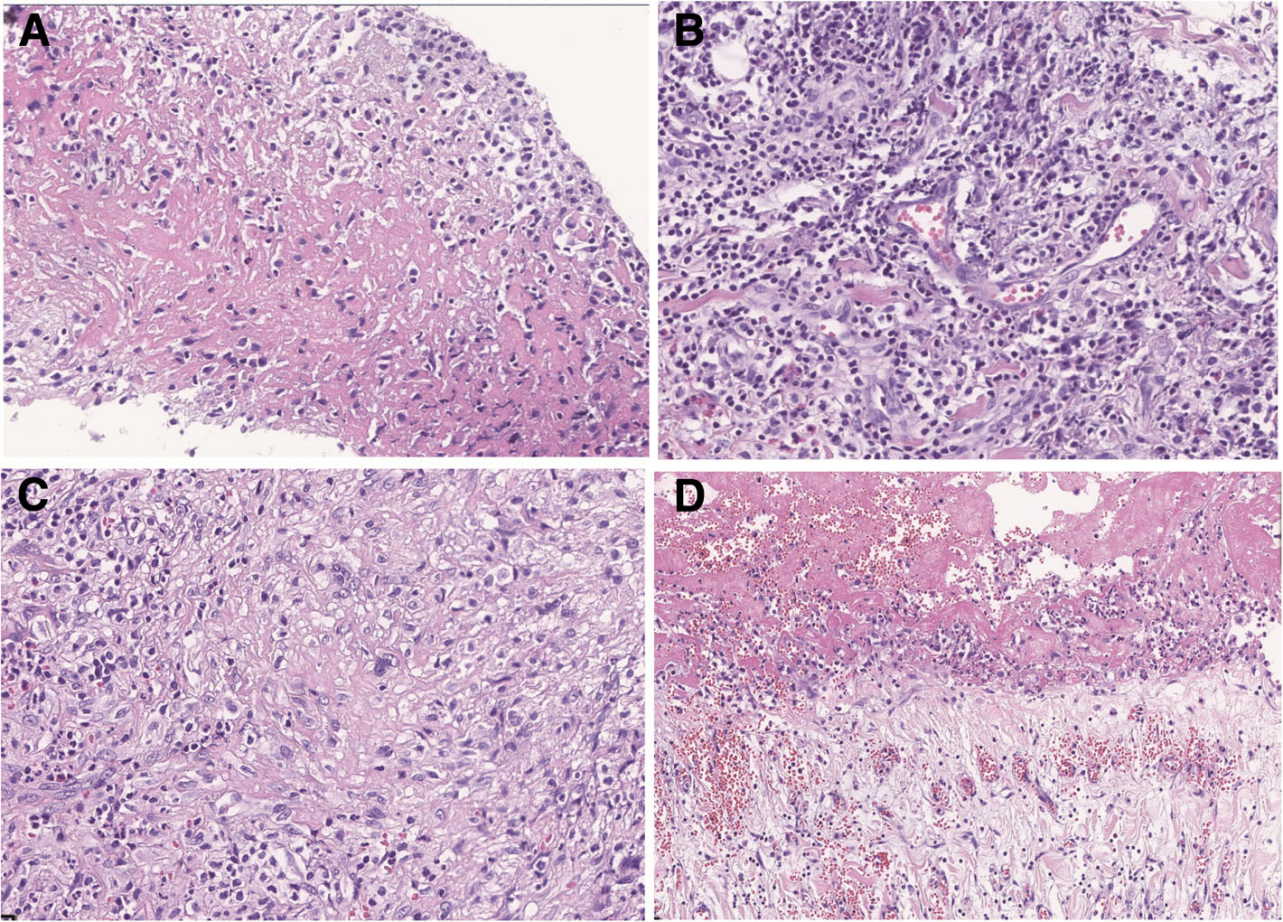
Pathological characteristics of the patient: **a** inflammatory cell infiltration, cellulose exudation and necrosis in clamped lung tissues; **b** epithelioid cells and multinucleated giant cells forming a granulomatous reaction in the aorta; **c** small vasculitis in the aorta; **d** cellulose exudation and inflammatory granulation tissue hyperplasia in the pericardium(haematoxylin–eosin stain, magnification × 200)

A 27-year-old male was admitted to hospital emergency due to 4 h of chest pain in 13 January 2016. In fact, he had suffered from fatigue after activity for more than 10 days and felt chest tightness and chest pain for 4 days before admission. The patient, a taekwondo trainee, had a healthy body, and his family history was unremarkable. Emergency aortic computed tomographic (CT) scan showed a thickened aortic wall, bilateral pleural effusion and pericardial effusion (Figs. 1a and b). Cardiac colour ultrasound suggested aortic hematoma or dissection (Fig. 2). On the basis of the condition and the results of auxiliary examination, the formation of aortic dissection should be considered. The next day, after excluding surgical contraindications, the patient was performed an emergency operation. Cardiac surgeons underwent ascending aortic replacement and aortic arch replacement. They developed postoperative comprehensive treatment measures, including anti-infection, adjustment of cardiac function, nutritional nerve and symptomatic supportive treatment, and the patient recovered well and was discharged after 15 days. The cause of aortic dissection was unclear, although the surgeons extracted arterial tissues and pericardial tissues and sent them for pathological examination. In fact, postoperative pathology of the aortic wall tissue showed aortitis, such as visible necrosis, granulation tissue hyperplasia and a large number of acute and chronic inflammatory cell infiltration (Figs. 3b, c and d). However, the surgeons and the pathologist at the time did not consider the cause of aortitis or aortic dissection due to GPA because of a lack of understanding of GPA-induced aortitis or aortic dissection. This event is one of the starting points of our study. We want to attract the attention of clinicians. GPA is also a common cause of aortitis and even aortic dissection.

On the first day of 2017, the patient was re-examined for aortic CT because of a 6-day fever and right chest pain. He showed a good prognosis in terms of aortic dissection after a review of aortic CT, but we found significantly increasing lesions in his lung, such as flake density increased shadow, less clear boundary, visible cavity and bronchial meteorology (Fig. 1c). The patient had no renal insufficiency and sinusitis but had eye damage with scleritis. The next day, the patient was readmitted to our hospital. At first, we considered pulmonary infection because of the combination of fever, haemogram, pulmonary shadow and cavitary lesions. Hence, we administered moxifloxacin. After 12 days, we reviewed chest CT again and found that the lung lesions became significantly heavier than before (Fig. 1d). Moreover, the patient still had intermittent fever, and the infection treatment was ineffective. At the same time, the patient developed conjunctivitis in the left eye and pain in the finger joints. Thus, we started to suspect pulmonary infection. We checked connective tissue disease-related indicators, such as ANCA, anti-nuclear antibody and immune indicators. The proteinase 3 (PR3)-ANCA (c-ANCA) level was 180 IU/mL, and the MPO-ANCA (p-ANCA) level was 10 IU/mL. Rheumatoid factor and anti-O experiments were positive, and anti-nuclear antibody spectrum was negative. We then performed a bronchoscopy, including brush biopsy, bronchoalveolar lavage and transbronchial lung biopsy. The pathological results suggested inflammatory cell infiltration, cellulose exudation and necrosis in clamped lung tissues (Fig. 3a). However, these pathological changes may be insufficient to diagnose GPA. We therefore carefully re-examined the pathological findings of the patient’s aorta and pericardium one year ago. Surgical biopsies obtained from the aorta and pericardium tissue showed that the epithelioid cells and multinucleated giant cells formed a granuloma (Fig. 3b), small vasculitis (Fig. 3c and Additional file 1: Figure S1 and Additional file 2: Figure S2) existed in the aorta and cellulose exudation and inflammatory granulation tissue hyperplasia were present in the pericardium (Fig. 3d).Combining with the two pathological results, clinical manifestations and laboratory tests, we invited a radiologist and a pathologist to perform a multidisciplinary discussion in the initial diagnostic assessment of the patient with suspected GPA. Finally, the diagnosis of GPA was established. Then, immunosuppressive therapy with *i.v.* steroids (methylprednisolone 40 mg twice daily) and cyclophosphamide 125 mg·day^− 1^ was initiated. The patient is currently followed up with the above treatment programs. We also adjusted the treatment program according to the patient’s disease progression. At present, the patient recovers well and is in stable condition.

## Discussion and conclusion

The patient was eventually diagnosed with GPA in accordance with the American College of Rheumatology criteria for GPA [9]. In detail, the patient suffered from multiple organ damage, including the lung, left eye and aorta; p-ANCA was positive; and pathological results from the aorta and pericardium tissue supported GPA.

GPA is one of the ANCA-associated systemic vasculitis of medium-sized and small blood vessels [4].The exact cause is unknown, but genetic predisposition, infections, environment or pharmacological agents may trigger an inflammatory response that involves the release of pro-inflammatory cytokines and ANCA [6]. GPA, rarely involving the artery, mainly accumulates medium-sized and small blood vessels. The most frequent findings of cardiac involvement in GPA are pericarditis and coronary vasculitis [8]. However, GPA with aortic dissection is very rare. A literature survey was performed in PubMed (up to 29 March 2019) by using the following key words: ‘*Wegener’s granulomatosis*’, ‘*granulomatosis with polyangiitis*’, ‘aorta’, ‘aortitis’, ‘dissection’ and ‘aneurysm’ in different combinations. Only cases with sufficient clinical data for analysis were reviewed. Finally, 15 case reports were included in the present study [10–24]. The main characteristics of 16 cases with aortitis and aortic dissection due to granulomatosis with polyangiitisare summarised in Table 1. We found 2 female patients in Spain and 14 male patients distributed in Japan (4 patients),the Netherlands (3 patients), Belgium (2 patients), South Korea (1 patient), Greece (1 patient), the United Kingdom (1 patient), American (1 patient) and China (1 patient). The age of onset ranged from 28 to 79. Moreover, almost all patients have positive ANCA. In addition, only three patients had a ruptured aorta, of which two died. Immunosuppressive agents were the main treatment, and the prognosis of surviving patients was good.

**Table 1:** Characteristics of patients with aortitis and aortic dissection due to granulomatosis with polyangiitis

First author /year	Gender/Age (y)	Country	Location	Manifestation	Antibodies	Aneurysmal symptoms	Duration of aneurysmal symptoms	Aneurysmal symptoms from GPA diagnosis	Therapy	Rupture	Outcome
Sieber/ 1990	Male/59	American	Aorta	Aortic aneurysm	c-ANCA positive	Abdominal pain	9 months	9 months before	Coronary artery bypass + steroid pulse + PSL + CY 3 mg/kg + plasmapheresis	No	Good
Fink/1994	Male/45	United Kingdom	Aorta	Aortitis	ANCA positive	Malaise and intermittent right abdominal pain	5 months	5 months after	Right ureterolysis + immunosuppressive therapy	No	Good
Blockmans/2000	Male/42	Belgium	Aorta	Aortic aneurysm + aortic dissection	c-ANCA 1: 1280, Antiproteinase-3157 AU/l	Abdominal pain	1 week	3 weeks after	Aortoiliac graft and high-dose steroids + CY 2 mg/kg/day	No	Good
Chirinos/2004	Female/50	Spain	Thoracic aorta	Thoracic aortic aneurysm	p-ANCA (1: 320) antimyeloperoxidase 440 U/ml	Abdominal pain with radiation to the back	2 weeks	Concomitant	mPSL pulse CY	Yes	Death
Carels/2005	Male/63	Belgium	Abdominal aorta	Abdominal aortic aneurysm	p-ANCA 1:80 and MPO 28 U/l	Low back pain, fever	2 months	Concomitant	Surgery +PSL 1 mg/kg + CY 2 mg/kg	No	Good
Minnee/2009	Male/51	Netherlands	Aorta	Aortic aneurysm	ANCA positive, anti-proteinase-3 antibodies > 530 kU/L	Lower back pain	2 months	Concomitant	Steroid pulse + PSL 1 mg/kg + CY 2 mg/kg/day	No	Good
Durai/2009	Male/33	Netherlands	Abdominal aorta	Abdominal aortic aneurysm	Antiproteinase-3 positive (> 1/10)	Abdominal discomfort in the upper abdomen	3 weeks	3 weeks after	Internal jugular vein graft + PSL+ CY	No	Good
Unlü/2011	Male/43	Netherlands	Infrarenal aorta	Infrarenal aortitis with aneurysm	NS	Abdominal pain and generalized malaise	1 week	11 years after	PSL + surgery	No	Good
Toda /2011	Male/79	Japan	Thoracic aorta	thoracic aortic aneurysm	Proteinase 3-ANCA 1180 EU	Back pain	8 months	8 months after	Steroid + PSL 60 mg + intravenous CY 300 mg	Yes	Death
Amos /2012	Male/64	Greek	Aorta	Aortitis	ANCA positive	Fever, intermittent pleuritic chest pain	4 weeks	Concomitant	CY 1.5 mg/kg/day and intravenous mPSL 500 mg/day	No	Good
Ohta /2013	Male/38	Japan	Thoracic aorta	Thoracic aortic aneurysm	ANCA positive(× 128)	Back pain and lossofconsciousness	NS	22 years after	Surgery (J-graft) + PSL 15 mg/day	Yes	Good
Miyawaki/2017	Male/60	Japan	Aorta	Aortitis	Proteinase 3-ANCA 1500 U/mL	Fever and cough	NS	26 years after	mPSL 1 mg/kg daily and CY 2 mg/kg daily	No	Good
Niimi/2018	Male/57	Japan	Abdominal aorta	Abdominal aortic aneurysm	Proteinase 3-ANCA 187 IU/ml	Back pain and fever	1 month	Concomitant	Steroid + PSL 55 mg + intravenous CY 500 mg	No	Good
Kim /2018	Male/58	South Korea	Ascending arta	Aortitis and intramural hematoma	ANCA positive	Mid-sternal pain	NS	Concomitant	steroid treatment	No	Good
Parperis/2019	Female/71	Spain	Aorta	Aortitis	p-ANCA positive, MPO antibody 159	Chronic headache	20 years	20 years after	PSL 60 mg daily and methotrexate 20 mg weekly with folic acid 1 mg daily	No	Good
Present case	Male/28	China	Aorta	Aortitis and aortic dissection	c-ANCA positive	Chest pain	4 h	1 years after	Aortoiliac graft + mPSL 40 mg twice daily and CY 2 mg/kg daily	No	Good

*c-ANCA* Proteinase 3 (PR3)-antineutrophil cytoplasmic antibody, *PSL* Prednisolone, *CY* Cyclophosphamide, *p-ANCA* Myeloperoxidase (MPO)-antineutrophil cytoplasmic antibody, *mPSL* Methylprednisolone, *MPO* Myeloperoxidase; NS: not stated

Considering our patient’s diagnostic process, we summarise some valuable experiences or lessons. Firstly, we should focus on the patient’s medical history and physical examination, and detailed medical history and physical examination often provide diagnostic evidence. Secondly, we should attach importance to the close relationship between clinical and pathological findings. Thirdly, we must pay attention to the relationship between clinical changes and treatment effects. We need to dynamically observe the diagnosis of disease and efficacy of consistency, and any observation that does not meet our physician’s expectations should not be ignored because these may be the only evidence to correctly diagnose the disease. Fourthly, the diagnosis of GPA requires information from many sources to be interpreted and integrated by clinicians. As we all know, a multidisciplinary team (MDT) is composed of healthcare workers from different disciplines who will share information and work interdependently. An MDT of pulmonologists, radiologists and pathologists for GPA diagnosis is important and highly recommended, which can later gradually become the mainstream model for clinical diagnosis and treatment. A rare disease with a rare manifestation is difficult to diagnose. We therefore focus on rare manifestations of rare diseases when we encounter problems in clinical diagnosis. We need to develop good clinical diagnostic thinking, especially in patients with incurable diseases.

In conclusion, we believe that GPA should be included in the systemic vasculitis that can give rise to aortitis and even aortic dissection. It is considered a cause of aortic dissection, which should not be ignored by clinicians. In the diagnosis of difficult diseases, especially systemic diseases, we should pay attention to rare presentations. We should also consider the relationship among clinical, pathologic and imaging data, and focus on the patient suffering from the disease.

## Additional files

Additional file 1: **Figure S1.** Pathological figure from the aorta to demonstrate the small vasculitis. (TIF 107 kb)

Additional file 2: **Figure S2.** Pathological figure from the aorta to demonstrate the small vasculitis. (TIF 1970 kb)

## Data Availability

The datasets used and/or analysed during the current study are available from the corresponding author on reasonable request.

## Abbreviations

ANCA: Anti-neutrophil cytoplasm antibody
CT: Computed tomographic
GPA: Granulomatosis with polangiitis
MDT: Multidisciplinary team
c-ANCA: Proteinase 3 anti-neutrophil cytoplasm antibody
WG: Wegener’s granulomatosis
