# Supplementary figures and images for: Case report of a 28-year-old man with aortic dissection and pulmonary shadow due to granulomatosis with polyangiitis
Source: BMC Pulm Med. 2019 Jul 8;19:122. doi: 10.1186/s12890-019-0884-9 (PMC6615146; doi:10.1186/s12890-019-0884-9)

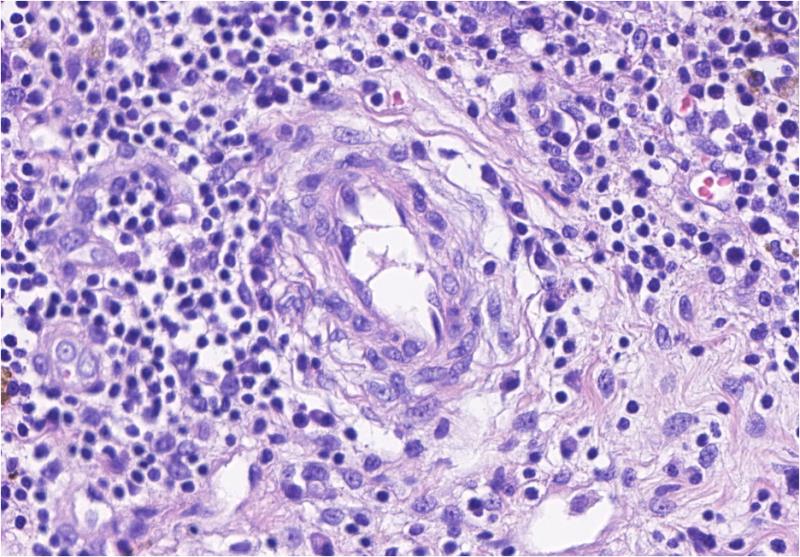

Supplement: Supplementary file 1 — Figure S1. Pathological figure from the aorta to demonstrate the small vasculitis. (TIF 107 kb) [file 12890_2019_884_MOESM1_ESM.tif]

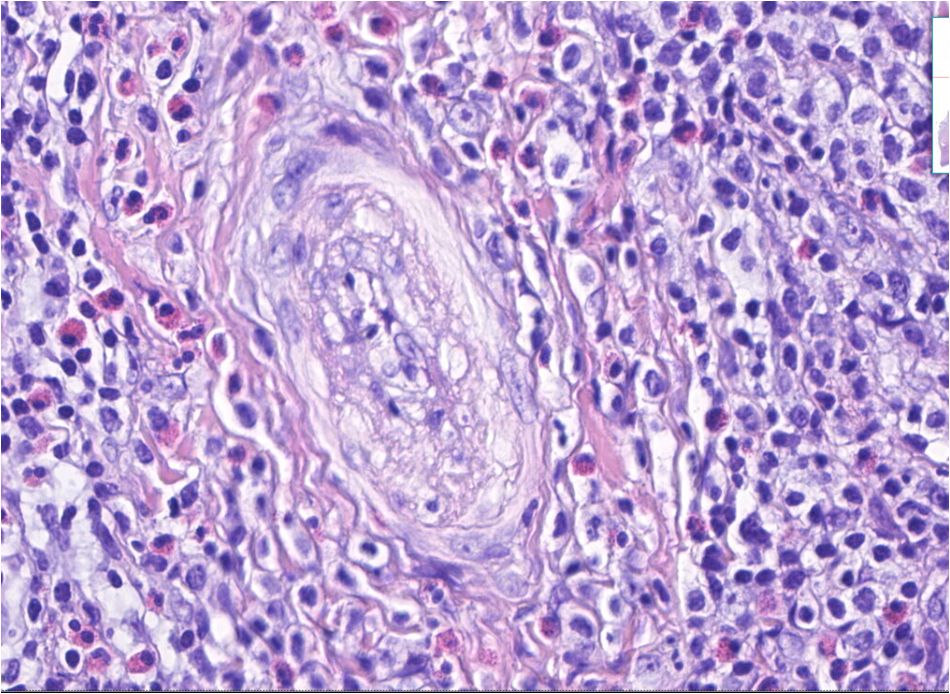

Supplement: Supplementary file 2 — Figure S2. Pathological figure from the aorta to demonstrate the small vasculitis. (TIF 1970 kb) [file 12890_2019_884_MOESM2_ESM.tif]
